# Synchronous Primary Pleural Mucosa-Associated Lymphoid Tissue Lymphoma and Pulmonary Squamous Cell Carcinoma in a Patient With Asbestos Exposure: A Case Report

**DOI:** 10.7759/cureus.93467

**Published:** 2025-09-29

**Authors:** Rie Irie, Toshiaki Kawai, Naoya Nakamura, Masayuki Okui, Hideki Orikasa

**Affiliations:** 1 Pathology, Nippon Koukan Hospital, Kawasaki, JPN; 2 Pathology, Kawasaki Municipal Hospital, Kawasaki, JPN; 3 Pathology, Tokai University School of Medicine, Isehara, JPN; 4 Thoracic Surgery, Kawasaki Municipal Hospital, Kawasaki, JPN

**Keywords:** asbestos, extranodal low-grade b-cell lymphoma, extranodal marginal zone malt lymphoma, pleural malt lymphoma, squamous cell carcinoma

## Abstract

Primary pleural extranodal marginal zone lymphoma of mucosa-associated lymphoid tissue (MALT) is extremely rare. We report the case of a Japanese man in his 70s who had a history of asbestos exposure and known bilateral pleural plaques and presented with dyspnea. Imaging studies revealed a pulmonary mass in the left lower lobe, and a biopsy confirmed squamous cell carcinoma of the lung. Positron emission tomography-computed tomography (PET-CT) incidentally revealed increased radiotracer uptake in the pleura between the third and fourth left ribs. The pleural mass was diagnosed as primary pleural MALT lymphoma on biopsy. To the best of our knowledge, this is the first reported case of the synchronous occurrence of primary pleural MALT lymphoma and pulmonary squamous cell carcinoma in a patient with a documented history of asbestos exposure.

## Introduction

Pulmonary mucosa-associated lymphoid tissue (MALT) lymphoma originating from bronchial MALT accounts for less than 1% of primary lung tumors. Pleural MALT lymphoma is exceedingly rare, with only 15 cases reported in the English literature to date [1-15]. To our knowledge, only one case each has been reported in a patient exposed to asbestos in the English and Japanese literature [1,15]. We report a case of synchronous primary pleural MALT lymphoma and pulmonary squamous cell carcinoma in a patient with asbestos exposure.

## Case presentation

**Figure 1:**
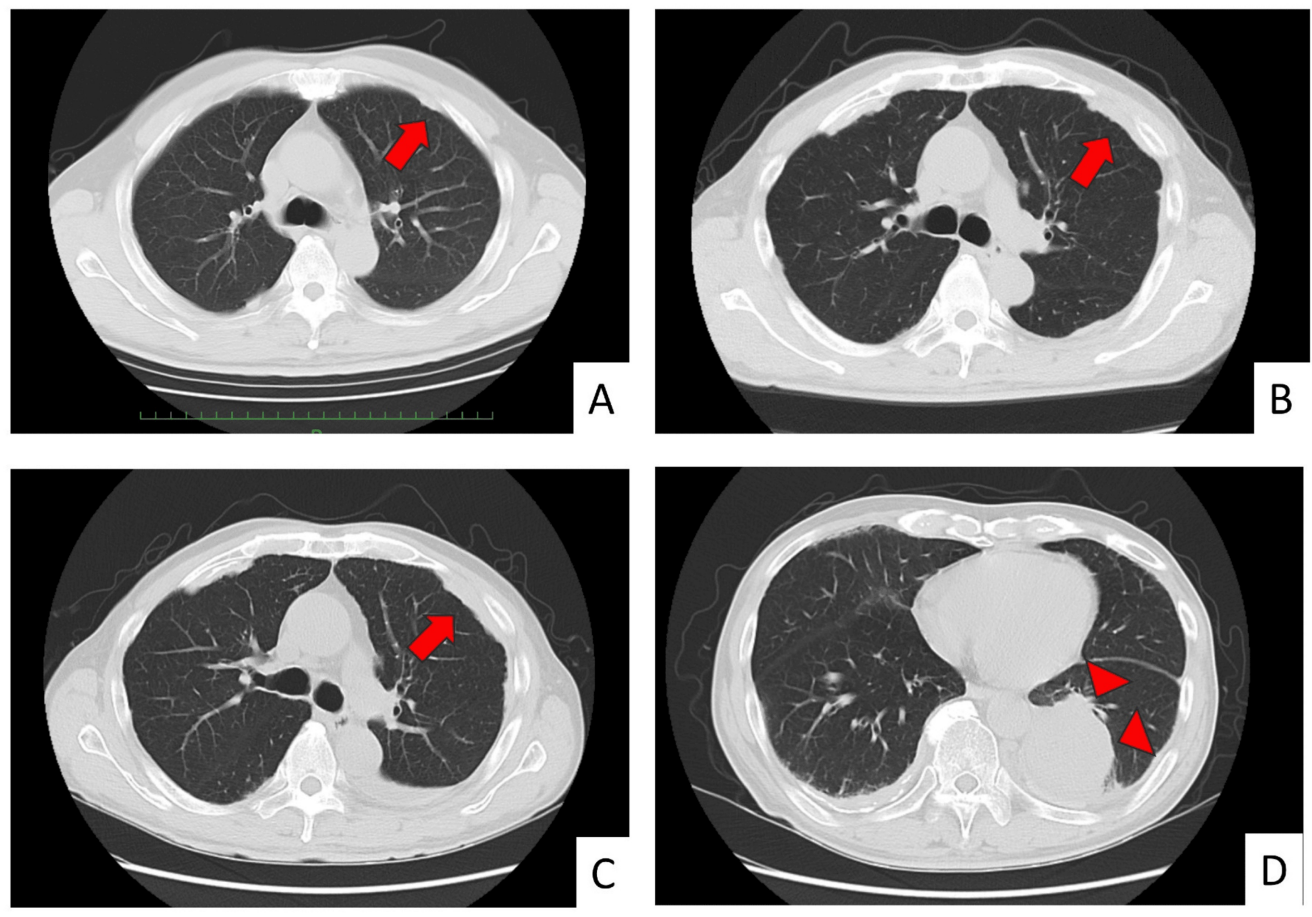
Chest computed tomography. (A) Twelve years prior (year X-12): a small nodule around the left third intercostal space (arrow). (B) Three years prior (year X-3): a pleural plaque between the left third and fourth intercostal spaces (arrow). (C) Year X: pleural thickening can be seen centered at the left third/fourth intercostal space (arrow). Compared to the lesions in the image from 12 years earlier, the lesion in the left third/fourth intercostal space shows a tendency to increase in size over time. (D) Year X (same as C): a mass measuring approximately 68 mm in the left lower lobe (arrowheads).

The patient was a man in his 70s with a history of smoking and occupational asbestos exposure during shipbuilding and building pipe installations for approximately 50 years. The patient was under outpatient follow-up for benign prostatic hyperplasia and showed bilateral pleural plaques on imaging (Figure 1).

Compared to the lesion observed through imaging 12 years earlier (Figure 1A), the lesion in the left third/fourth intercostal space showed a tendency to increase in size over time (Figure 1B, 1C). The patient was hospitalized for exertional dyspnea, and chest computed tomography (CT) revealed a mass sized approximately 68 mm in the left lower lobe of the lung (Figure 1D). On admission, laboratory findings revealed a leukocyte count of 12,820/μL, a mildly elevated CRP of 3.93 mg/dL, a lactate dehydrogenase (LDH) of 253 IU/L, and a soluble IL-2 receptor level of 2,153 U/mL. Tumor markers were elevated, with carcinoembryonic antigen (CEA) at 5.4 ng/mL and cytokeratin fragment antigen (CYFRA) at 5.9 ng/mL. The T-SPOT.TB test was negative.

**Figure 2:**
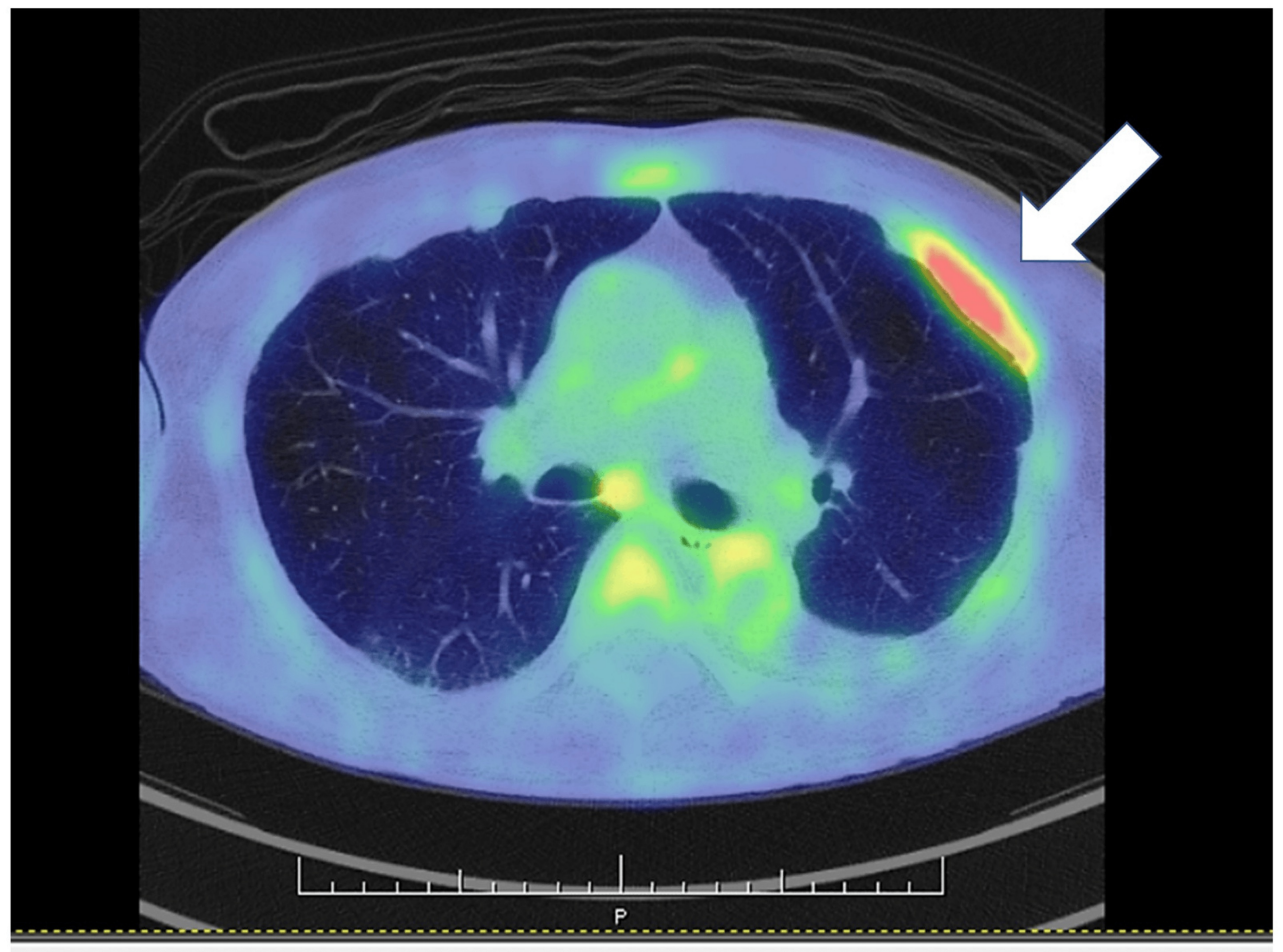
Positron emission tomography-computed tomography. Increased uptake (arrow), corresponding to the area of pleural thickening in the left third/fourth intercostal space (SUVmax: 4.89→5.77). SUVmax: maximum standardized uptake value

A transbronchial lung biopsy confirmed squamous cell carcinoma. Whole-body evaluation and positron emission tomography-computed tomography (PET-CT) for staging revealed focal uptake limited to the pleura between the left third and fourth ribs, in addition to the known lung tumor (Figure 2).

The lesion, measuring approximately 58×15 mm, was confined to the visceral pleura, with no evident invasion into the parietal pleura. No continuity with the mass identified in the lower lobe of the left lung was noted. Pleural biopsy was performed under the suspicion of either the dissemination of lung cancer or malignant pleural mesothelioma. The biopsy revealed low-grade B-cell lymphoma with no evidence of metastatic squamous cell carcinoma or malignant mesothelioma. As the lung cancer was diagnosed as stage IV, with hepatic metastasis, systemic chemotherapy was initiated as the primary treatment. Three months after the initiation of therapy, the patient remains alive.

Histopathological findings

Transbronchial Lung Biopsy

**Figure 3:**
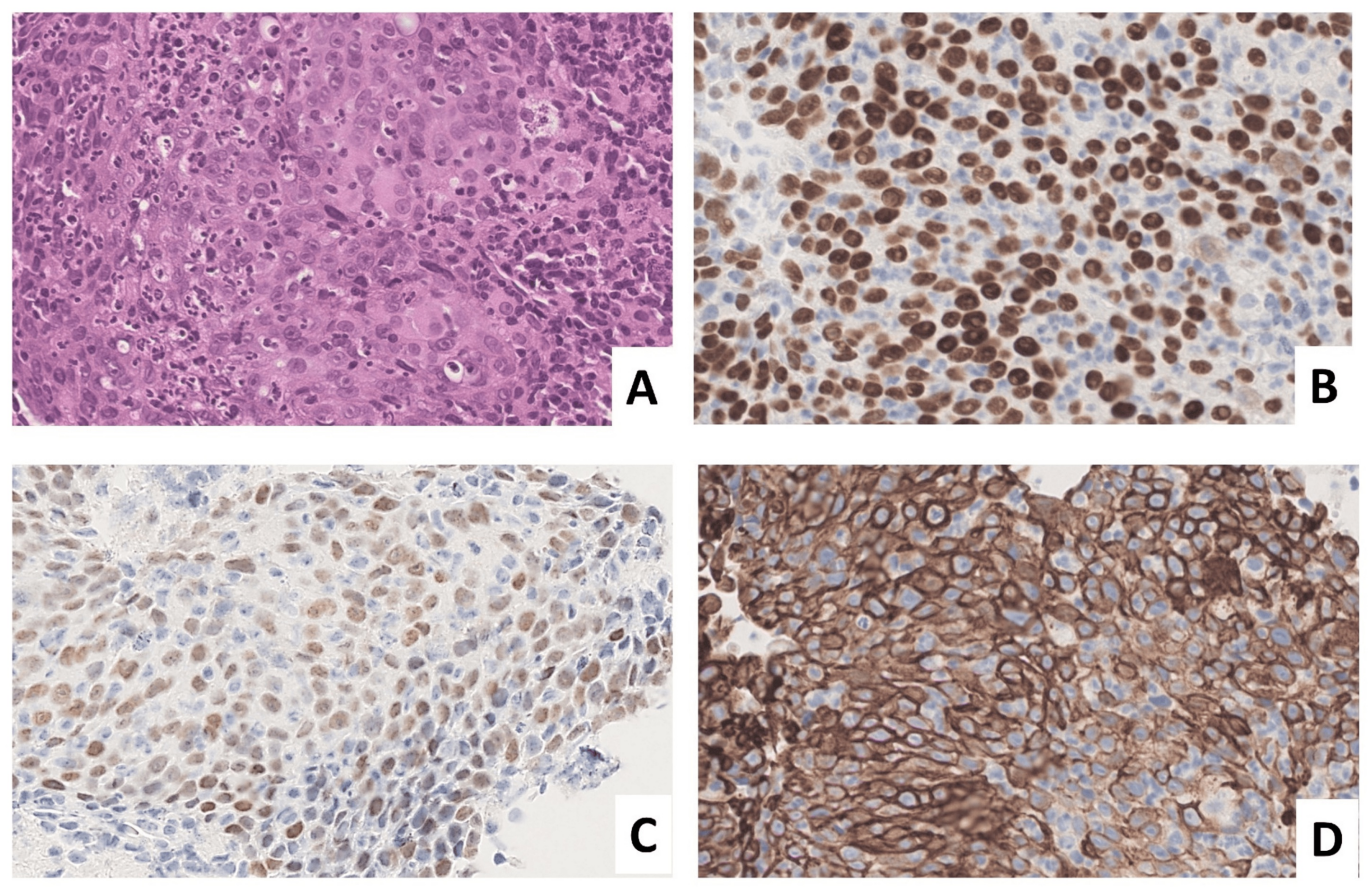
Transbronchial lung biopsy from segment B10 of the left lung. (A) Tumor cells with large, atypical nuclei and prominent nucleoli proliferate in nests (hematoxylin and eosin stain). The tumor cells are positive for p40 (B), p63 (C), and CK5/6 (D). CK5/6: cytokeratin 5/6

Tumor cells exhibited large atypical nuclei with prominent nucleoli and formed nests accompanied by surrounding inflammatory infiltrates. Although definite keratinization was not observed, immunohistochemical staining revealed that the tumor cells were positive for p40, p63, and cytokeratin 5/6 (CK5/6) and negative for thyroid transcription factor-1 (TTF-1) and napsin A, consistent with poorly differentiated squamous cell carcinoma (Figure 3).

No asbestos bodies were detected through iron staining.

Pleural Biopsy

**Figure 4:**
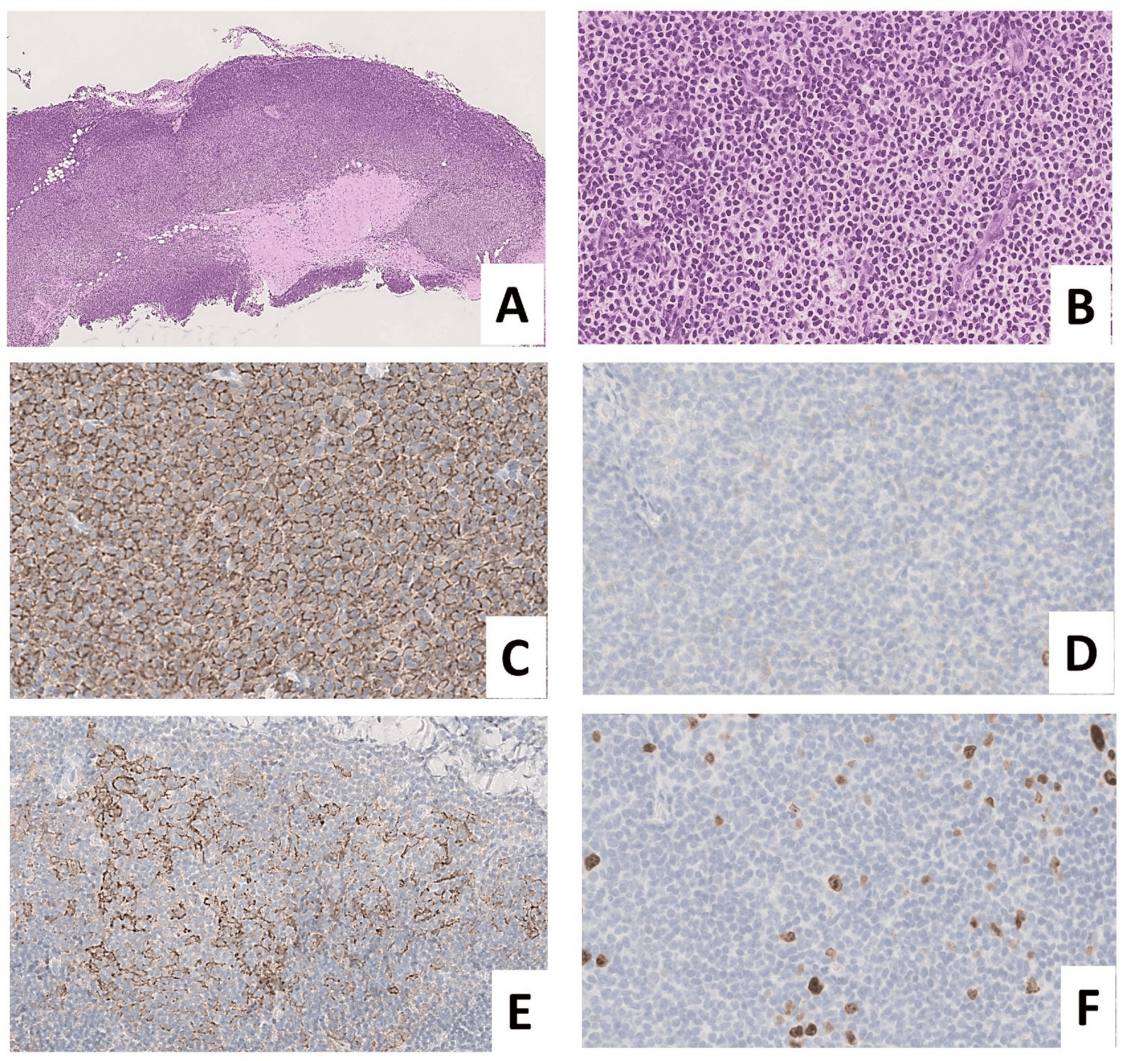
Pleural biopsy. (A) Dense, band-like lymphocytic infiltration is seen along the pleura. Background pleural tissue showed thickening and hyalinization (hematoxylin and eosin stain, ×4). (B) Small- to medium-sized tumor cells with mild nuclear atypia proliferate densely. No definite follicular architecture is observed (hematoxylin and eosin stain, ×40). Atypical lymphoid cells are CD20-positive (C) and CD10-negative (D). (E) The distribution of follicular dendritic cells is irregular, and well-demarcated germinal center structures are not evident (CD21). (F) Ki-67 labeling index is 2%-3%. CD: cluster of differentiation

A dense proliferation of medium-sized lymphoid cells with minimal atypia was observed. Some follicle-like structures without clear germinal centers were observed. Immunohistochemically, the lymphoid cells were positive for cluster of differentiation 20 (CD20), CD79a, B-cell lymphoma 2 (BCL2), and BCL6 and negative for CD3, CD5, CD10, and cyclin D1. CD21 did not exhibit a distinct follicular dendritic cell network (Figure 4).

The Epstein-Barr virus-encoded small RNA in situ hybridization (EBER-ISH) was negative. The background pleura showed thickening and hyalinization (Figure 4A) without atypical mesothelial proliferation, and limited PET-CT uptake led to the diagnosis of primary pleural MALT lymphoma. A fluorescence in situ hybridization (FISH) analysis of the pleural tumor revealed no break-apart signal points for *MALT1* (data not shown).

## Discussion

The association between asbestos exposure and lung cancer is well-established. Asbestos is recognized as an independent risk factor for primary lung cancers, including adenocarcinoma, squamous cell carcinoma, and small cell carcinoma, among which adenocarcinoma is the most common. In this case, although no asbestos bodies were found in the biopsy specimen and the patient had a history of smoking, the possibility that asbestos exposure contributed to the development of squamous cell carcinoma cannot be excluded.

A strong correlation exists between asbestos exposure and malignant pleural mesothelioma. Pleural plaques are not precursor lesions and do not transform into mesothelioma; rather, they are markers of asbestos exposure and are associated with an increased risk of developing malignant mesothelioma. This patient had been under observation for pleural plaques detected approximately 12 years prior to the diagnosis of squamous cell carcinoma. PET-CT performed for cancer staging revealed uptake in the region corresponding to the pleural plaques. Based on these findings, the clinical suspicion of pleural dissemination or mesothelioma led to a pleural biopsy, which unexpectedly revealed malignant lymphoma. Diffuse large B-cell lymphoma is the most common type of primary pleural lymphoma, and Epstein-Barr virus (EBV)-associated pyothorax-associated lymphoma is well-known. In this case, the histological findings were consistent with low-grade MALT lymphoma, and the EBER-ISH result was negative. The patient had no history of chronic pyothorax.

MALT lymphoma is most frequently observed in the gastrointestinal tract, thyroid, and salivary glands; in the lungs, it commonly arises from the bronchial mucosa. These sites typically involve a background of chronic inflammation, such as *Helicobacter pylori* infection, autoimmune thyroiditis, or autoimmune disease. In this case, the lymphoma coincided with the location of pre-existing pleural plaques, suggesting that the chronic inflammation associated with these plaques may have contributed to lymphomagenesis.

To date, only 15 cases of primary pleural MALT lymphoma have been reported in the English literature, with approximately half of them originating from Japan. Among these, only one case each from the United Kingdom and Japan involved asbestos exposure (Table 1) [1,15].

**Table 1:** Reported cases of primary pleural MALT lymphoma NA, not available; CHOP, cyclophosphamide, hydroxydaunorubicin, oncovin, and prednisolone; R-CHOP, rituximab-CHOP; M, male; F, female; FISH, fluorescence in situ hybridization; MALT, mucosa-associated lymphoid tissue; PD-L1, programmed death-ligand 1; SCC, squamous cell carcinoma

Author (year)	Age/sex	Nationality	Chief complains/symptoms	Smoking	Asbestos exposure	FISH *MALT1*	Treatment	Complications
Ahmad et al. (2003) [1]	59/M	England	Dyspnea and chest pain	(-)	(+)	NA	Chlorambucil	None
Ahmad et al. (2003) [1]	49/M	England	Dyspnea	(-)	(-)	NA	Chlorambucil and prednisolone	Tuberculosis
Hirai et al. (2004) [2]	72/M	Japan	Dyspnea and pleural effusion	NA	NA	NA	Thoracotomy and chemotherapy	None
Mitchell et al. (2006) [3]	47/M	Canada	Fever, chest pain, and pleural effusion	(+)	(-)	NA	Thoracotomy	Pyothorax (acute)
Gomyo et al. (2007) [4]	67/F	Japan	Dyspnea and pleural effusion	(-)	(-)	(+)	Chemotherapy	IgM paraproteinemia
Kawahara et al. (2008) [5]	79/M	Japan	Back pain and pleural effusion	(+)	(-)	NA	Thoracotomy	None
Motta et al. (2010) [6]	74/M	Italy	Cough and weakness	(-)	(-)	NA	Rituximab	None
Barahona et al. (2011) [7]	54/M	Spain	None (pleural mass)	(-)	(-)	NA	NA	None
Nakatsuka et al. (2012) [8]	86/M	Japan	None (pleural plaque)	(+)	(-)	NA	NA	Metal exposure
Okamoto et al. (2019) [9]	39/M	Japan	None (pleural mass)	NA	NA	(+)	NA	None
Ben Saad et al. (2019) [10]	64/M	Tunisia	Cough and dyspnea	NA	NA	NA	CHOP and radiotherapy	Rheumatoid arthritis
Paul et al. (2020) [11]	62/M	USA	Dyspnea and weight loss	NA	NA	NA	Chemotherapy	Chylothorax
Wang et al. (2022) [12]	54/M	China	Cough and shortness of breath	(-)	NA	NA	R-CHOP	None
Urano et al. (2023) [13]	57/F	Japan	None (abnormal shadow on chest X-ray)	NA	NA	(+)	Rituximab	Rheumatoid arthritis
Onatsko et al. (2024) [14]	88/F	USA	Cough and shortness of breath	(-)	(-)	NA	Rituximab	Adenocarcinoma of the lung
This case (2025)	74/M	Japan	Dyspnea	(+)	(+)	(-)	None (PD-L1 for lung cancer)	SCC of the lung
Inoue et al. (2014) [15]	76/M	Japan	Pleural effusion	(-)	(+)	NA	CHOP and rituximab	None

Regarding the link between asbestos exposure and hematological malignancies, a few reports suggest that asbestos may be a risk factor for leukemia and lymphoma [16,17]. While asbestos exposure and smoking are well-known risk factors for lung malignancies, only two cases of synchronous pulmonary MALT lymphoma and squamous cell carcinoma have been reported [18,19]. Of these, the case reported by Guo et al. involved a patient with a history of smoking but without documented asbestos exposure [19].

Due to the rarity of primary pleural MALT lymphoma, the role of asbestos exposure remains uncertain. However, in the present case, the lymphoma arose from an area of pre-existing pleural plaques, suggesting a possible relationship between asbestos-induced chronic pleural inflammation and the development of MALT lymphoma.

Primary pleural MALT lymphoma is a rare entity; as summarized in Table 1, most of the previously reported cases in the English literature have originated from Japan. In the Japanese literature, more than 10 cases can be identified, including those published as abstracts only. Diffuse large B-cell lymphoma associated with chronic inflammation (pyothorax-associated lymphoma) was first described in Japan as a subtype of B-cell lymphoma. This condition is known to occur following artificial pneumothorax therapy for tuberculous pleuritis or pulmonary tuberculosis, and cases have predominantly been reported from Japan. In one case reported by Ahmad et al., primary pleural MALT lymphoma was associated with tuberculosis [1]. Although no explicit description was provided in previous Japanese reports, in elderly patients, a possible history of tuberculosis or tuberculous pleuritis cannot be completely excluded.

Furthermore, fluid overload-associated large B-cell lymphoma has also been frequently reported in Japan. Thus, several subtypes of B-cell lymphoma are relatively more common in Asia, particularly in Japan. In EBV-negative cases, however, the underlying reasons remain unclear, and the further accumulation of cases together with comprehensive studies, including genetic analyses, is warranted. In our case, diagnostic tests for tuberculosis were negative at the time MALT lymphoma was identified, and lymphoma cells were negative for EBER-ISH.

The histopathological examination of the pleural lesion in the present case revealed features consistent with those of MALT lymphoma. However, FISH analysis did not detect* MALT1* gene splitting. The *t(11;18)(q21;q21)/BIRC3::MALT1* fusion has been reported in approximately 40% of pulmonary MALT lymphomas. Remstein et al. reported that the frequency of chromosomal translocations may vary depending on the anatomical site of MALT lymphoma [20]. To date, only three cases of pleural MALT lymphoma with reported FISH findings have been described in the English literature: Gomyo et al. [4] identified a *MALT1* gene split, Okamoto et al. [9] reported trisomy 18 involving *MALT1*,* *and Urano et al. [13] documented a *t(14;18)(q32;q21.3)/IGH-MALT1* translocation. Notably, no case reports published in original Japanese articles have included the FISH analysis of primary pleural MALT lymphoma. Further case studies are needed to elucidate the frequency of *MALT1* abnormalities in pleural MALT lymphoma.

## Conclusions

To the best of our knowledge, this is the first reported case of synchronous primary pleural MALT lymphoma and pulmonary squamous cell carcinoma in a patient with a history of smoking and asbestos exposure. Further accumulation of cases is needed to clarify the potential association between asbestos exposure and hematological malignancies, including MALT lymphoma.

In addition, the relatively high incidence of certain B-cell malignant lymphomas, including primary pleural MALT lymphoma, in Japan underscores the necessity of further research. Future studies, particularly those addressing potential genetic predispositions, will be essential to elucidate the pathogenesis and to advance our understanding of the regional characteristics of these lymphomas.
